# Lessons and Untapped Potential of Smartphone-Based Physical Activity Interventions for Mental Health: Narrative Review

**DOI:** 10.2196/45860

**Published:** 2024-03-15

**Authors:** Emily E Bernstein, Emma C Wolfe, Brynn M Huguenel, Sabine Wilhelm

**Affiliations:** 1 Department of Psychiatry Massachusetts General Hospital Boston, MA United States; 2 Department of Psychiatry Harvard Medical School Boston, MA United States; 3 Department of Psychology University of Virginia Charlottesville, VA United States

**Keywords:** smartphone, digital health, exercise, physical activity, mental health, depression, anxiety, mobile phone

## Abstract

**Background:**

Physical activity has well-known and broad health benefits, including antidepressive and anxiolytic effects. However, only approximately half of Americans meet even the minimum exercise recommendations. Individuals with anxiety, depression, or related conditions are even less likely to do so. With the advent of mobile sensors and phones, experts have quickly noted the utility of technology for the enhanced measurement of and intervention for physical activity. In addition to being more accessible than in-person approaches, technology-driven interventions may uniquely engage key mechanisms of behavior change such as self-awareness.

**Objective:**

This study aims to provide a narrative overview and specific recommendations for future research on smartphone-based physical activity interventions for psychological disorders or concerns.

**Methods:**

In this paper, we summarized early efforts to adapt and test smartphone-based or smartphone-supported physical activity interventions for mental health. The included articles described or reported smartphone-delivered or smartphone-supported interventions intended to increase physical activity or reduce sedentary behavior and included an emotional disorder, concern, or symptom as an outcome measure. We attempted to extract details regarding the intervention designs, trial designs, study populations, outcome measures, and inclusion of adaptations specifically for mental health. In taking a narrative lens, we drew attention to the type of work that has been done and used these exemplars to discuss key directions to build on.

**Results:**

To date, most studies have examined mental health outcomes as secondary or exploratory variables largely in the context of managing medical concerns (eg, cancer and diabetes). Few trials have recruited psychiatric populations or explicitly aimed to target psychiatric concerns. Consequently, although there are encouraging signals that smartphone-based physical activity interventions could be feasible, acceptable, and efficacious for individuals with mental illnesses, this remains an underexplored area.

**Conclusions:**

Promising avenues for tailoring validated smartphone-based interventions include adding psychoeducation (eg, the relationship between depression, physical activity, and inactivity), offering psychosocial treatment in parallel (eg, cognitive restructuring), and adding personalized coaching. To conclude, we offer specific recommendations for future research, treatment development, and implementation in this area, which remains open and promising for flexible, highly scalable support.

## Introduction

### Background

In the 21st century, anxiety and depression have been among the top 25 causes of global disease burden [1]. The COVID-19 pandemic has only intensified the rising prevalence as well as the personal and societal impacts of these disorders [2,3]. As there are simply not enough mental health professionals to meet this need [4,5], alternative interventions—both preventive and curative—are urgently needed. Targeting physical activity is a clear opportunity. Before the pandemic, more than 1 in 4 adults reported sitting for >8 hours per day [6], and this number rose to >40% during the pandemic [7]. Both of these statistics are likely underestimated [8]. Prolonged sedentary behavior, or extended time spent awake with minimal energy expenditure [9], is associated with more severe anxiety and depression as well as higher odds of developing related disorders [10-14]. In contrast, decades of research have demonstrated that regular physical activity is associated with numerous positive psychological outcomes [15-17]. Cross-sectionally, individuals who engage in regular exercise—a subset of physical activity that involves planned, structured, and repetitive bodily movement intended to improve or maintain fitness [18]—report fewer and less severe symptoms of anxiety and depression [19,20], greater positive affect and well-being [21], less stress [22], and lower rates of anxiety and depressive disorder diagnoses [23-25]. At the individual level, people report feeling better on days when they exercise [26-28].

### Exercise as an Intervention for Depression and Anxiety

Prospective data support regular exercise as a potent population-level *prevention* tool, significantly lowering the risk of developing anxiety and depressive disorders [25,29,30]. Encouragingly, even small amounts of physical activity may have an enormous impact on mental health [31,32]. For example, an estimated 12% of new cases of depression could be prevented if the entire population exercised for just 1 hour per week [33]. In addition, among individuals presenting with diagnosable levels of symptomatology, systematically increasing exercise behavior is therapeutic [33-36].

Exercise appears to enhance emotional flexibility, or a person’s ability to self-regulate under stress [37-40]. Physiologically, individuals who exercise more regularly show faster heart rate recovery following stressors than their peers who exercise less, and individual bouts of exercise can mitigate the hypothalamic-pituitary-adrenal axis, heart rate, and blood pressure reactivity to acute stress [39,41-43]. Physical activity can also increase the production of brain-derived neurotrophic factors, which are neurobiological changes that are understood to increase resilience [44]. These effects are mirrored in reports of exercise bolstering emotional recovery following stressors, enhancing coping self-efficacy, and mitigating the impact of rumination and other emotion regulation deficits on prolonging distress [34,39,42,45,46]. Furthermore, exercise benefits physical health targets that share bidirectional relationships with mental health, such as better sleep and cardiometabolic health [47-51]. Critically, positive treatment effects have been found for directly alleviating anxiety [52-55] and depressive [56-58] disorders as well as related and frequently comorbid conditions such as posttraumatic stress disorder [59] and obsessive-compulsive disorder [60,61]. Importantly, research has replicated the benefits of physical activity (ie, reducing psychiatric symptoms) in samples of people with severe mental illness, such as schizophrenia [62]. Similarly, physical activity and other health-related behaviors (eg, sleep hygiene) are considered to be an integral component of treatment for bipolar disorder [63]. Exercise has also been successfully used to augment the effects of other validated psychosocial treatments such as cognitive behavioral therapy (CBT) [64-67].

Despite the broad knowledge that regular physical activity is physically, cognitively, and emotionally beneficial, only approximately half of Americans meet even the minimum exercise recommendation of 150 minutes per week of moderate-intensity or equivalent physical activity [68]. Individuals with anxiety, depression, or related conditions are even less likely to do so [69,70]. They are also more likely than peers without mental health disorders to exhibit elevated sedentary behavior [71,72]. Thus, although acceptable and efficacious tools exist to help individuals meaningfully change their behavior and improve psychiatric symptoms, there is a large gap between the evidence and real-world implementation. Few clinicians include physical activity as an explicit treatment target or use it as an intervention tool [73-75]. Moreover, the larger barriers to treatment within our health care system remain, including the inaccessibility of treatment due to the acute shortage of qualified clinicians; stigma; and patients’ difficulty with travel, timing, and the cost of regular appointments [76].

### Promise of Digital Platforms for Promoting Physical Activity

With the advent of mobile sensors and phones, experts have quickly noted the ability of technology to expand the reach of evidence-based psychiatric care; overcome the aforementioned barriers by providing treatment flexibly; and begin reducing long-standing disparities in treatment access, response, and dropout [77-79]. This could also be an efficient, scalable method of promoting increased physical activity among adults with or at risk of anxiety and depressive disorders.

Digital solutions show strong early benefits for activity measurement and intervention in nonpsychiatric populations. In fact, leveraging technology to measure and increase physical activity was an official recommendation from the National Heart, Lung, and Blood Institute and National Institute on Aging “Influences on sedentary behavior/Interventions to reduce sedentary behavior” joint workshop [80]. First, measuring behavior via mobile sensor is validated and widely used [81-84]. Similarly, people’s tendency to carry their phones with them throughout the day allows for more accurate monitoring of physical activity and related progress. The ease of use and, therefore, precision of such technologies (wearable and smartphone-based sensors) is an important boon for research and treatment as self-report measures of activity typically result in underreporting [81-84].

Second, delivering treatment in part or fully via mobile phones is effective in increasing physical activity in nonpsychiatric populations [85-87]. This parallels broader findings that apps can effectively promote other health behaviors (eg, improved nutrition, smoking cessation, and medication adherence) [88,89]. Currently, 97% of Americans have a mobile phone, and an estimated 85% have a smartphone [90]. Although these numbers are lower in certain populations, such as those with serious mental illness (wherein an estimated 85% own a mobile phone and 60%-70% own a smartphone), the ubiquity of smartphones allows for the promotion of behavior change in real time and with a wider array of individuals [91-94]. Inactivity frequently occurs out of conscious awareness or choice due to people’s attention being fixated elsewhere (eg, watching television or taking the elevator at work). As such, personal devices can unobtrusively enhance awareness of behavior, which itself can promote increased activity [95]. Furthermore, technologies can deliver notifications in the moment to interrupt passive episodes while also providing tools to increase activity when individuals are most likely to take action [96]. Mobile app–based physical activity interventions can also gamify exercise to enhance enjoyment, which is a key mechanism for long-term engagement in physical activity [97,98]. In-the-moment enjoyment not only promotes regular exercise but is also in itself beneficial for mental health, contributing to the success of broader interventions such as behavioral activation. Overall, digital interventions are promising as they are low risk (ie, typically focus on reducing sitting and increasing light activity), can be deployed without a clinician, and can be used in the context of a patient’s daily life. Ultimately, research conducted thus far in the general population supports mobile technologies as valid, accessible, and effective methods of promoting physical activity and reducing sedentary behavior.

### Current Objective

Although it is reasonable to extrapolate that physical activity interventions could be implemented via smartphone in a similarly feasible, acceptable, and effective manner in psychiatric populations or for psychiatric targets, this remains an open question. High-quality trials of in-person exercise programs for mental health often unintentionally include components beyond the activity itself that are potentially therapeutic, such as regular, structured, and supervised sessions [65]. In other words, as part of most exercise programs, participants also receive regular social engagement or support, face-to-face time with a professional, instruction and demonstration of target behaviors, and guidance with behavioral scheduling or activation, all of which may enhance the therapeutic benefits of physical activity. However, the remote and asynchronous nature of technology-based interventions may provide a different experience from that of in-person programs, and thus, the impact may also differ. On the other hand, the aforementioned benefits of digital interventions, such as their ability to increase accessibility, lower logistical barriers to engagement, and enhance self-awareness while also promoting behavior change in real time, may boost response and, thus, lead to comparable—or even stronger—effects than face-to-face trials. As a result, it cannot be assumed that face-to-face physical activity programs or digital programs designed for other populations (eg, medical) will translate when delivered via smartphone or to a new population.

In this study, we explored the potential of physical activity interventions, as delivered (at least in part) via smartphone, to improve mental health in psychiatric populations. As this topic remains relatively new, we also considered available evidence on these tools to address mental health symptoms in nonpsychiatric populations. Specifically, we highlighted in which populations these tools have been tested; what outcomes have been evaluated (eg, acceptability, behavior change, and symptom change); and how strategies and tools have (or have not) been tailored to individuals with depression, anxiety, or related concerns. The goal was to provide a narrative overview and specific recommendations for future research on smartphone-based physical activity interventions for psychological disorders or concerns.

## Methods

### Literature Search

To provide a narrative overview of this emerging research area, we searched for articles that (1) described or reported an intervention intended to increase physical activity or reduce sedentary behavior; (2) included an emotional disorder, concern, or symptom as an outcome measure; (3) described or reported an intervention delivered entirely or in part via a smartphone app; and (4) were published in English and in peer-reviewed journals. Example search terms include “smartphone,” “smartphone application,” “mobile application,” “mobile app,” “digital mental health,” “app-based,” “app-assisted,” “mobile phone,” “ehealth,” “digital,” “mobile,” “exercise,” “physical activity,” “sedentary,” “sedentary behavior,” “physical inactivity,” “depression,” “dysthymia,” “mood,” “anxiety,” “phobia,” “trauma,” “posttraumatic stress,” “obsessive compulsive disorder,” “post-traumatic stress,” “obsessive-compulsive disorder,” “stress,” “emotional disorder,” “emotional problem,” “well-being,” “wellness,” “affective disorder,” “OCD,” “PTSD,” “MDD,” “GAD,” “mental health,” and “mental illness.” Web-based database (PubMed, Google Scholar, and Cochrane) searches and additional manual searches (eg, searching the reference sections of articles identified through database searches) were conducted up to March 2022. Records were initially reviewed by one coauthor; in cases of uncertainty about appropriateness for this review, records were reviewed in full by 2 additional coauthors and discussed until a consensus was reached.

### Data Review

We attempted to extract the following information, where available, from each paper: sample size, inclusion criteria, demographics of the sample, primary aim, trial design (eg, randomized controlled trial), treatment duration, technology used, other interventions used (ie, in addition to physical activity), outcome measures (eg, primary and secondary measures of physical activity), results, treatment components or behavior change strategies, adaptations for mental health, and inclusion of coaching.

## Results

### Overview

In taking a narrative lens, we drew attention to the type of work that has been done and used these exemplars to discuss key directions to build on. Table 1 provides a summary of the included articles.

**Table 1:** Summary of studies investigating the impact of digital physical activity interventions on mental health symptoms.

Study, year	Sample size, N	Population studied	Intervention used	Duration	Physical activity outcome	Primary psychiatric outcome	Primary medical outcome
Aguilera et al [99], 2020^a^	N/A^b^	Adults with diabetes and a score of >5 on the PHQ^c^	Apps: DIAMANTE to track data and deliver adaptive learning algorithm (active only) and HealthySMS to send messages (active and control)	6 months	Steps	Depressive symptoms (PHQ-8^d^)	HbA_1c_^e^ levels (blood glucose)
Broers et al [100], 2019	557	Adults diagnosed with hypertension, symptomatic heart failure, or coronary artery disease	Wearable (Fitbit) activity tracker, wearable (Beddit) sleep tracker, app (Moves) GPS tracker, app (Careportal) home monitoring system	6 months	Steps; physical activity level (combined length of active periods and step count)	Anxiety (GAD-7^f^) and depression (PHQ-9^g^)	N/A
Damschroder et al [101], 2020	357	Web-based confirmation of veteran status	App (Stay Strong) and wearable (Fitbit Charge 2)	12 months	Active minutes per week; steps	N/A	N/A
Edney et al [102], 2020	444	Adults currently completing <150 minutes of MVPA^h^ per week	App (Active Team) and wearables (pedometer and Zencro TW64S)	3 months	Daily minutes of MVPA	Symptoms of anxiety, depression, and stress (DASS-D^i^)	N/A
García-Estela et al [103], 2021^a^	N/A	Spanish-speaking adults with MADRS^j^ score of >1	App (IDEApp) and wearable (smartband)	8 months	SIMPAQ^k^; functional exercise capacity (6MWT^l^ and 1-min sit-to-stand test); short Borg CR-10^m^ Scale	Depressive symptoms (PHQ-9); well-being (WHO-5^n^)	Global functioning (SF-36v2^o^)
Guo et al [104], 2020	300	Adults who are HIV-seropositive with elevated depressive symptoms	App (WeChat) and Run4Love program (adapted CBSM^p^ course and physical activity promotion) delivered through the WeChat app	3 months	Chinese version of the GPAQ^q^	Depressive symptoms (CES-D^r^)	N/A
Haufe et al [105], 2020	314	Adults with metabolic syndrome	Wearable (activity monitor; Forerunner 35; Garmin)	6 months	Freiburger Questionnaire on Physical Activity; steps	Anxiety severity and depression severity (HADS^s^)	Change in metabolic syndrome severity; health-related quality of life (SF-36^t^)
Kim et al [106], 2021	21	Adults aged >46 years diagnosed with PD^u^ or atypical parkinsonism conditions and regular participation in a PD exercise program at least once a week	App (researcher-created physical activity app)	8 weeks	Total exercise calculated by multiplying the frequency and duration for all exercises; subjective exercise scale (Borg 6-20 scale); IPAQ^v^	Depression (Geriatric Depression Scale–Short Form)	PDQ-39^w^
Lin et al [107], 2020^a^	N/A	Physically inactive adults with a musculoskeletal diagnosis (*ICD-10*^x^) and in rehabilitation following inpatient clinic treatment	App (MoVo)	12 months	BSA^y^, sport activity and movement activity subscales	Depression (PHQ-9); anxiety (GAD-7)	Brief Pain Inventory
Ma et al [108], 2015^a^	N/A	Adult participants who were obese and experiencing depression	Wearable (Fitbit) and app or website (MyFitnessPal)	12 months	Minutes of physical activity logged on MyFitnessPal	Depression (SCL-20^z^)	Changes in BMI
Nadal et al [109], 2021^a^	N/A	Adult users with mild to moderate depression who were assigned to iCBT^aa^ treatment for depression	Wearable (smartwatch; Mood Monitor watch app)	8 weeks	Smartwatch activity data	Depression (PHQ-9), anxiety (GAD-7), and functional impairment (WSAS^ab^)	N/A
Park et al [110], 2021	60	Adults with a history of cardiovascular disease who were within 2 weeks of completing cardiac rehabilitation	Wearable (Fitbit Charge 2) and apps (Movn and Fitbit)	2 months	Steps; 6MWT; self-reported physical activity	Quality of life (QLESQ^ac^); depression (PHQ-9)	N/A
Puszkiewicz et al [111], 2016	13	Adults with a diagnosis of breast, prostate, or colorectal cancer who had finished primary curative treatment	App (GAINFitness)	6 weeks	Physical activity (GLTEQ^ad^)	Health and quality of life outcomes (EQ-5D); well-being (FACT-G^ae^); anxiety and depression (HADS)	Cancer-related fatigue (FACIT^af^); sleep quality (PSQI^ag^)
Puterman et al [112], 2021	334	Adults with a score of 1-3 on the L-CAT^ah^ who were cleared to exercise	App (Down Dog suite of apps—HIIT^ai^ and yoga)	6 weeks	Sessions of yoga or HIIT completed; minutes of yoga or HIIT completed	Depressive symptoms (CESD)	N/A
Skrepnik et al [113], 2017	172	Adults with osteoarthritis eligible to receive the hylan G-F 20 injection	Wearable (Jawbone UP24) and app (OA GO)	90 days	Steps	VAMS^aj^	Changes in sleep captured by the wearable activity monitor
Stephens et al [114], 2022	15	Youth (aged 11 to ≤18 years) with MS^ak^ and a disability rating of <4 on the EDSS^al^ and attending a pediatric MS and neuroinflammatory disorder clinic	App (Atomic)	12 weeks	Physical activity measured via accelerometry; time spent in MVPA and sedentary activities; aerobic fitness, musculoskeletal strength, and walking endurance	Depression (CES-DC^am^)	N/A
Teychenne et al [115], 2021	62	Mothers 3-9 months post partum, insufficiently active, and experiencing heightened depressive symptoms	App (smartphone app and web forum)	12 weeks	Self-reported physical activity; accelerometer-assessed physical activity and sedentary behavior	Depressive and anxiety symptoms (unstandardized questionnaires)	N/A
Wilczynska et al [116], 2020	N/A	Adults with or at risk of type 2 diabetes	App (eCoFIT)	20 weeks	N/A	Depressive and anxiety symptoms (PHQ-9 and GAD-7)	Social support, self-efficacy, nature relatedness, and perceived sleep quality
Wong et al [117], 2021	79	Adults with moderate depressive symptoms	App (Lifestyle Hubl)	8 weeks	Physical activity level (IPAQ)	Depressive and anxiety symptoms (PHQ-9 and GAD-7)	Insomnia (ISI^an^); health-related quality of life; health-promoting behaviors (HPLP-II^ao^); functional impairment (SDS^ap^)

^a^Published protocol.

^b^N/A: not applicable.

^c^PHQ: Patient Health Questionnaire.

^d^PHQ-8: Patient Health Questionnaire-8.

^e^HbA_1c_: hemoglobin A_1c_.

^f^GAD-7: Generalized Anxiety Disorder-7.

^g^PHQ-9: Patient Health Questionnaire-9.

^h^MVPA: moderate to vigorous physical activity.

^i^DASS-D: Depression Anxiety Stress Scales: Depression Subscale.

^j^MADRS: Montgomery–Åsberg Depression Rating Scale.

^k^SIMPAQ: Simple Physical Activity Questionnaire.

^l^6MWT: 6-Minute Walk Test.

^m^CR-10: Borg Category-Ratio scale.

^n^WHO-5: World Health Organisation–Five Well-Being Index.

^o^SF-36v2: 36-Item Short Form Health Survey version 2.

^p^CBSM: cognitive behavioral stress management.

^q^GPAQ: Global Physical Activity Questionnaire.

^r^CES-D: Center for Epidemiologic Studies Depression Scale.

^s^HADS: Hospital Anxiety and Depression Scale.

^t^SF-36: 36-Item Short Form Health Survey.

^u^PD: Parkinson disease.

^v^IPAQ: International Physical Activity Questionnaire.

^w^PDQ-39: Parkinson Disease Questionnaire-39.

^x^*CD-10: International Statistical Classification of Diseases, Tenth Revision*I.

^y^BSA: Movement and Sport Activity Questionnaire.

^z^SCL-20: Symptom Checklist Depression Scale.

^aa^iCBT: internet-based cognitive behavioral therapy.

^ab^WSAS: Work and Social Adjustment Scale.

^ac^QLESQ: Quality of Life Enjoyment and Satisfaction Questionnaire.

^ad^GLTEQ: Godin Leisure-Time Exercise Questionnaire.

^ae^FACT-G: Functional Assessment of Cancer Therapy–General.

^af^FACIT: Functional Assessment of Chronic Illness Therapy.

^ag^PSQI: Pittsburgh Sleep Quality Index.

^ah^L-CAT: Stanford Leisure-Time Categorical Activity Item.

^ai^HIIT: high-intensity interval training.

^aj^VAMS: visual analog mood scale.

^ak^MS: multiple sclerosis.

^al^EDSS: Expanded Disability Status Scale.

^am^CES-DC: Center for Epidemiologic Studies Depression Scale for Children.

^an^ISI: Insomnia Severity Index.

^ao^HPLP-II: Health-Promoting Lifestyle Profile-II.

^ap^SDS: Sheehan Disability Scale.

### Who Was Included in This Work?

Overall, a review of the literature revealed that little work has been done to test the impact of smartphone-based physical activity interventions on increasing physical activity or reducing mental health symptoms in psychiatric or at-risk populations. Most related trials with clinical populations have been conducted in the area of medicine, with studies investigating the effects of physical activity—encouraged through smartphone- and wearable-based interventions—on physical health conditions (eg, diabetes [99,116], obesity [86,108], cancer [111,118], cardiovascular issues [100,110], and multiple sclerosis [114]). Physical activity is well established as a means of facilitating rehabilitation following serious illness or injury, as well as mitigating the progression of chronic health conditions [119-121]. For example, adults with Parkinson disease who used a minimally supported, customizable home-based exercise app for 8 weeks doubled their amount of weekly exercise (minutes) while also increasing the intensity of such exercise [106]. Similarly, engagement with smartphone-based physical activity interventions led to increased strenuous exercise among adults with cancer [111] as well as increased step count for those with cardiac issues [100,110] and for youths with multiple sclerosis [114]. These changes are noteworthy as medical illness or disease can serve as a barrier to engaging in health-promoting behaviors [122] despite the knowledge that such behaviors can stabilize or even improve such medical conditions [123].

In contrast, the practice of formally integrating exercise into *mental* health care is relatively new and not established in current standards of care. This is mirrored by the disproportion of extant research examining digital tools for increasing physical activity in medical versus psychiatric populations. When mental health targets were examined, they were largely included as secondary or exploratory outcomes and frequently framed in relation to coping with the medical concern of interest [99,100,104-108,110,111,113,114]. We only identified 16% (3/19) of the studies that specifically recruited individuals with psychiatric symptoms, and all (3/3, 100%) were focused on individuals with depressive symptoms [103,109,117]. The most common mental health outcomes were depression, anxiety, general quality of life, and emotional well-being [99,100,102-110,112,114-117]. Specifically, subclinical depressive concerns were the most frequently investigated psychiatric target, followed by subclinical anxiety [100-105,112,115-117].

The impact of smartphone-based physical activity interventions is yet to be investigated explicitly for individuals diagnosed with depressive or anxiety disorders, let alone other mental health conditions, including serious mental illness. Furthermore, although wide age brackets were represented across the studies, with average ages ranging from teenagers to older adults, men and non-White individuals were underrepresented. As research progresses in this space, it will be imperative to include the experiences and perspectives of adults with clinical levels of psychiatric concerns as well as diverse backgrounds and identities.

### Do Smartphone-Based Physical Activity Interventions Benefit Mental Health?

Owing to the limited available data; small samples comprising mostly White Western, educated, industrialized, rich, and Democratic women with subclinical depression or anxiety; and heterogeneity of outcomes measured, it is difficult to conclude whether and to what extent existing smartphone-based physical activity interventions benefit mental health. However, with these caveats in mind, we aimed to synthesize the available evidence in the following sections.

#### Feasibility and Acceptability

There is encouraging evidence that smartphone-based physical activity interventions could be feasible and acceptable for psychiatric populations. However, supporting data were primarily collected in samples of individuals with elevated depressive symptoms or who were at risk of depression rather than in explicitly clinical samples or among individuals with other prominent mental health concerns. One study of postnatal women at risk of depression found *low* engagement with the digital aspects of a 12-week multicomponent physical activity intervention (home exercise equipment and a physical logbook combined with a motivational smartphone app and a web-based social support forum) [115]; however, other studies reported more positive participant response and engagement. For example, retention in smartphone interventions for physical activity tended to be high compared to that in other types of digital health interventions—one systematic review found that completion rates of digital mental health interventions ranged from 1% to 28% [124]. In a study of adults with diabetes, retention in a 20-week digital physical activity intervention (an app that allowed participants to use workout circuits, set goals, monitor progress, and learn cognitive and behavioral strategies) was of >70% [99]. Similarly, compliance with a suite of high-intensity interval training (HIIT) and yoga apps during a 6-week intervention was strong in a community sample with elevated depressive symptoms. More than half of the participants in the yoga and HIIT+yoga group and 40% in the HIIT group continued completing the recommended 4 sessions per week by the end of the trial [112]. Compliance and satisfaction ratings were comparably high when physical activity promotion was combined with cognitive behavioral stress management via a WeChat intervention in a group of adults with HIV and elevated depressive symptoms [104].

These findings are consistent with those of the larger literature showing that digital physical activity interventions tend to be well received by participants [85,125,126]. Public interest is already high, with physical activity and fitness apps dominating the mobile health space. Notably, a 2018 systematic review of the experience of adults who used mobile interventions to promote physical activity highlighted important themes to be considered for future design—self-reported engagement was most enhanced by the availability of social features, prompts, goal setting, personalization or customization, and gamification but was limited by low technological literacy, preference for coached apps, and a desire for social support [127].

#### Change in Psychiatric Symptoms

The impact of smartphone-based physical activity interventions on psychiatric symptoms was far more mixed. Some studies observed resultant improvements in symptoms. In one study that included patients specifically recruited for having elevated symptoms of depression and anxiety, patients experienced a reduction in depression and anxiety scores following a 6-month exercise intervention (150 minutes of moderate physical activity per week with individual recommendations given via a smartphone app) as compared to a waitlist control. However, it is notable that this intervention did not test a smartphone-based physical activity intervention in isolation but, instead, combined it with nutritional counseling and the option of receiving exercise recommendations through personal meetings or by phone instead of an app [105]. Among adults from the general population with low physical activity scores, using a publicly available suite of exercise apps for 6 weeks significantly improved depressive symptoms compared to a waitlist control [112]. Psychiatric improvements were also observed in patients with medical comorbidities. For example, in a study of adults with obesity using a smartphone-based physical activity app (eCoFIT), depression symptom severity improved after 20 weeks [116]. Finally, in a study of older adults with Parkinson disease, using a mobile app to access and customize a home-based exercise program for 8 weeks led to reduced depression symptoms and improved quality of life [106].

However, other studies reported null effects. For example, among patients with a history of cardiovascular disease, a smartphone-based intervention including motivational prompts and educational messages did not yield significant changes in depressive symptoms from baseline to 2 months [110]. Furthermore, a trial of patients with cancer using a tailored physical activity smartphone app (which included workout videos, spoken instructions, and push notifications) did not observe changes in depression, anxiety, or quality of life after 6 weeks [111]. In a study testing an app-based physical activity program for youth with multiple sclerosis (including personalized coaching and promotion of aerobic fitness, musculoskeletal strength, and walking endurance), there was no change in depression levels over 12 weeks [114]. Furthermore, a meta-analysis of studies examining digital physical activity interventions in cancer survivors found that none of the included studies were successful in improving depression or anxiety [118]. In the general population, one study similarly did not find significant differences in depression, anxiety, stress, or well-being after 3 or 9 months of using an app (Active Team) and wearable pedometer [102].

These results should be interpreted cautiously for 2 reasons. First, there is the confound of potential floor effects—as none of these trials were designed to address questions about mental health, symptom levels at study start were typically already low, thus reducing investigators’ abilities to identify possible effects. In addition, studies varied widely in their evidence of behavior change, including outcome measures (eg, minutes of activity, number of sessions completed, exercise intensity, and fitness level; see Table 1 for detailed information on this variance) and the use of objective versus subjective reports, which are known to be discrepant [82-84]. In other words, if an intervention did not produce meaningful physical activity changes, it would be unlikely that downstream emotional changes would occur.

### How Have Smartphone-Based Physical Activity Interventions Been Tailored to Individuals With Depression, Anxiety, or Other Psychiatric Concerns?

To date, interventions generally have not been tailored to the specific needs or presentations of individuals with mental health concerns. This is unsurprising as most technology-based physical activity trials have not been designed to target mental health. However, some studies have integrated components that specifically address psychological well-being. One adaptation that is low effort but high return is adding psychoeducation about the relationship between physical activity and mental health. For example, a recent trial for adults with mild to moderate depressive symptoms devoted the first in-person group session to discuss the relationship between depression and exercise to complement the personalized exercise program, smartphone app, and wearable device they received [103].

The second adaptation observed in the literature is offering concurrent psychotherapy-based tools. In some cases, psychotherapeutic content was interspersed with the physical activity intervention; for example, in a trial for adults living with HIV and depression in China, both an exercise promotion intervention and cognitive behavioral stress management course were delivered as multimedia messages through the WeChat app [104]. Relatedly, in a study of the eCoFit app for adults with or at risk of type 2 diabetes, short cognitive behavioral tasks (“FitMind Challenges”) were integrated throughout the program [116]. Examples of FitMind Challenges included motivational strategies, relaxation, cognitive restructuring, social support, and problem-solving. In other cases, the approaches were delivered in parallel. In one trial, adults who were overweight and experiencing depression received a 7-step problem-solving therapy via a workbook in addition to live lifestyle coaching, at-home video lessons, the MyFitnessPal app, and a Fitbit for monitoring [108]. In another study, SilverCloud’s guided internet-based CBT program for depression was the primary intervention, with smartwatch-based monitoring of sleep, steps, and mood added to promote greater awareness of the relationship between health behaviors and mood, thereby independently encouraging positive lifestyle changes [109].

A third but largely unexplored avenue is the inclusion of personalized or tailored messaging. This is an opportunity for coaches or other support persons to address barriers that may be specific to the experience of someone with mental health concerns (eg, navigating social anxiety to go to the gym and restructuring depressive thoughts). In one open trial of a physical activity app for youth with multiple sclerosis, coaches were trained in social cognitive theory for behavior change as well as motivational interviewing [114].

Although few digital physical activity interventions have been designed or modified to specifically affect mental health, many have been designed using evidence- and theory-based behavior change strategies that are ripe for implementation in psychiatric contexts. Indeed, the most successful interventions are based on behavioral theory [73,127]—explicitly stated or not—such as the transtheoretical model [128], the theory of planned behavior [129], self-determination theory [130], and social cognitive theory [131]. Social cognitive theory is most often cited given its emphasis on internal, external, and social factors that reinforce learning and contribute to sustained change [131]. Targeting self-efficacy, self-regulation, and social support to engender meaningful, lasting behavior change aligns strongly with principles of psychotherapy as well. Digital physical activity interventions have also experimented with numerous evidence-based behavior change techniques, including goal setting and review, action planning, regular feedback, self-monitoring of behavior, instruction and demonstration of how to perform a new behavior, graded tasks, prompts and cues, and social rewards, to name a few [132-134]. Interventions integrating multiple behavior change strategies are more successful than those that rely on one (eg, self-monitoring or reminders alone [133]). Considering how such strategies could be adapted for individual presentations (eg, those with clinical levels of dysregulation) should be a priority for future iterations of these programs.

Furthermore, technology-driven techniques may uniquely (or at least more strongly than traditional treatments) engage key mechanisms of behavior change. For example, these tools can promote self-awareness. As people tend to keep their devices close to them throughout their daily lives, wearable and mobile platforms can provide objective, continuous monitoring and feedback related to behavioral patterns such as physical activity [102,108]. In addition, these approaches can enhance a person’s likelihood of changing their behavior by lowering the cognitive burden involved in initiating physical activity. Strategies include delivering content more flexibly (eg, when it is most convenient for a participant to engage or in doses of their choosing); modeling target behavior via written, image, or video instructions that can be reviewed on demand or infinite times; or tailoring activity suggestions to a person’s present context (eg, suggesting at-home activities on rainy days). This may be particularly meaningful for psychiatric audiences as depression and anxiety are associated with attention and memory deficits that can interfere with information processing and learning [135-138]. In addition, in-the-moment rewards and other gamification or reinforcement features could be particularly useful early on [139,140] as individuals with depression and anxiety may not experience initial sessions of exercise as intrinsically gratifying or mood boosting as others do; for example, depression is characterized by deficits in reward processing and motivation [141], and anxiety sensitivity and social anxiety can blunt positive responses or promote avoidance [142,143].

Finally, personal devices may allow for more consistent, flexible social support throughout an intervention. Social support is an established, evidence-based behavior change technique that promotes physical activity [126,144-146]. Smartphone-based physical activity interventions provide a range of avenues for social connection, such as texting with a coach [101,107], access to an web or app-based discussion forum [115], and creation of virtual “teams” [102]. Critically, although social media has been frequently incorporated as a means of facilitating connection, participant reactions have been mixed, and it may not be optimal for psychiatric populations [126]. In general, social support appears to boost engagement when it is perceived to facilitate emotional support, provide tips from peers, enhance motivation, foster social comparison or competition [126,147,148]. How to best leverage social support and social media for psychiatric populations requires nuanced future study.

## Discussion

### Principal Findings

The primary aim of this narrative synthesis was to examine the status of smartphone-based physical activity interventions for mental health and understand how they have and have not been tailored to or evaluated in psychiatric populations. Ultimately, the literature is limited and difficult to synthesize owing to the high heterogeneity across the studies in terms of sample selection; study design; outcomes of feasibility, acceptability, and efficacy; and degree of tailoring. To date, mental health outcomes have typically been secondary or exploratory within trials focused on medical outcomes (eg, diabetes management) and, when included, have had a narrow focus on measures of depression, anxiety, and general well-being in nonclinical populations. As a result, this review relied significantly on research focusing on medical populations to explore how smartphone-based physical activity interventions could be used to impact mental health outcomes and to infer how they may be used in psychiatric populations. Furthermore, although extant studies have included diversity of age, the samples in the included studies comprised mostly White and female individuals, thus reducing the generalizability of the already limited findings.

The feasibility and acceptability of these interventions for subclinical and at-risk populations are encouraging and suggest that digital physical activity programs may be similarly well received among individuals above diagnostic thresholds. The available data on psychiatric outcomes were mixed. However, it is difficult to draw meaningful conclusions given the limited data; high heterogeneity of intervention approach and target behavior; and lack of standardization in measurement and reporting of use, engagement, and behavior change, as well as the elevated risk of floor effects given the subclinical samples. These inconclusive psychiatric outcomes may also be related to a lack of tailoring of smartphone-based physical activity interventions to the specific needs of those presenting with mental health concerns. The existing tailoring included basic psychoeducation about physical activity as a treatment, adding concurrent psychotherapy-based tools, and including personalized or tailored messages. There was no standardization or evidence base for how this tailoring was applied. The upshot is that many of the papers included in this review presented interventions that were already built around established, evidence-based behavior change strategies, which suggests that psychotherapeutic tailoring could be efficiently integrated into existing smartphone-delivered physical activity interventions. In general, effective physical activity interventions use many of the same fundamental behavior change strategies commonly found in psychotherapy, such as education, goal setting, self-monitoring, graded tasks, engaging social support, and motivational interviewing [149,150].

Taken together, the primary barrier to advancing the use of smartphone-based physical activity interventions in mental health care is the absence of evidence. The need for research in this area has been highlighted in other reviews as well [151,152]. To construct a more consistent, evidence-based foundation for intervention development, we outline several avenues for future research.

### Recommendations for Tailoring Physical Activity Interventions to Psychiatric Populations

More research is needed to better understand *how* existing smartphone interventions can be tailored to fit the needs of psychiatric populations. The following are example adaptations rather than an exhaustive list. One likely critical step is to provide users with psychoeducation early on about the ways in which physical activity can be used to affect psychological health, such as improving mood and reducing anxiety. This should involve making explicit connections between health behaviors (eg, exercise), mental health symptoms, and emotion regulation so that users can better appreciate the bidirectional links between these areas of well-being. Including even a brief text summary of the literature or treatment rationale could likely augment the effects [153,154]. In fact, there is evidence with depression treatment that physical activity interventions lacking such psychoeducation or treatment rationale do not lead to robust clinical changes and can worsen dropout rates [155]. In contrast, attending to the mental and emotional benefits of exercise, particularly the acute or immediate impact on affect or resilience, can further enhance mood and motivation to continue exercising [35,156]. Highlighting these benefits and encouraging users to monitor such positive changes could improve sustained engagement and clinical response. Technology may be particularly helpful for this; apps, for example, can provide in-the-moment reminders through push notifications to attend to one’s affect or visual feedback of a user’s pretest-posttest change in self-reported mood with exercise. Furthermore, digital tools could provide information about the impact that mental health symptoms may have on program engagement. This can help users recognize that it is normal and expected for symptoms such as fatigue, anxiety sensitivity, or low motivation to serve as barriers to physical exercise and can proactively help users engage in related problem-solving.

Another compelling feature to test is the incorporation of modules or content that specifically address mental health concerns or symptoms. For example, technology-based physical activity interventions aimed at improving anxiety symptoms would benefit from including evidence-based skills such as cognitive restructuring and exposure practices. These approaches can be used to identify and challenge maladaptive beliefs that anxiety symptoms such as a racing heart are dangerous (known as anxiety sensitivity [157]), design a more graded exercise plan, and use activity as an interoceptive exposure by allowing patients to experience and tolerate those feared sensations [158]. Furthermore, CBT skills can be incorporated to address exercise-related social anxiety, such as integrating exposures (eg, walking with a friend or going to a gym first at off-peak hours) and challenging associated negative expectations (eg, “I won’t be able to keep up with my friend and they will judge me”). Meanwhile, a digital physical activity intervention aimed at helping people with depression could include skills consistent with behavioral activation, such as tracking the relationship between mood and activities (including physical activity); intentionally adding new behaviors such as gardening, walking, or going to the gym to their weekly schedule; and generating more flexible approaches to regular movement. Furthermore, cognitive skills can be used for participants to identify and evaluate negative thoughts about themselves or the program (eg, “I can never stick to my goal of going for walks, so what’s the point”) in terms of their accuracy or utility. In this vein, equipping coaches with some knowledge of common mental health symptoms to look out for, destigmatize, and address could enhance outcomes.

In addition, coaches are understood to bolster digital interventions in general by providing further psychoeducation or resources, personalizing content or skill use, and answering questions. Given the known relationships between mental illness and both inactivity and chronic medical conditions, even with basic mental health knowledge, coaches could perform these roles better. It is also important to recognize that digital or physical activity interventions may not be the appropriate or most effective level or type of care for all individuals experiencing mental health concerns. As such, tools should include information for users on the signs or symptoms that may indicate that pursuing psychotherapy could be beneficial as well as resources for doing so.

Finally, physical activity promotion tools could be integrated to augment existing treatments. Currently, there are a number of well-established treatments for psychiatric disorders, such as CBT and mindfulness. CBT has extensive research support as the gold-standard treatment for a range of disorders, including depression and anxiety [159]. This treatment integrates both behavioral and cognitive skills, such as tracking and scheduling activities as well as evaluating and challenging maladaptive thoughts, to reduce the severity and impact of symptoms. Mindfulness—or the purposeful, nonjudgmental awareness of the present moment [160]—is increasingly included in “third wave” interventions to reduce psychiatric symptoms [161] as well as increase well-being, such as positive affect and quality of life [162,163]. Recently, a dominant focus of digital mental health innovation has been translating these gold-standard psychotherapies to digital platforms. There is now a strong foundation of evidence supporting the feasibility, acceptability, and efficacy of delivering these treatments through both face-to-face and digital means [164-166].

Thus far, the development of digital interventions for physical activity and for mental health has largely occurred separately. However, their concurrent delivery provides promising initial evidence [111,118]. This parallels in-person trials demonstrating that increasing physical activity strengthens psychotherapy outcomes [167,168]. The next step in this line of research is to more formally integrate the 2 or even develop technologies in which both sets of skills are delivered within a single coherent platform. For example, in a study conducted by Wilczynska et al [116], adults with diabetes used the eCoFit app, which integrated guided workouts, goal setting, and cognitive behavioral skills. Some of the commercially available apps targeting mental health have already begun moving in this direction as well. The mindfulness-based app Headspace has recently incorporated a suite of video- and audio-guided exercises that help users engage in activities such as stretching, dancing, and yoga. Within these integrated platforms, it will be important to explicitly link the mental health and physical activity content rather than presenting them side by side as distinct intervention pathways.

### Recommendations for Developing and Testing Physical Activity Interventions for Populations With Psychiatric Disorders

#### Overview

More research is also needed to understand *to what degree* smartphone interventions require tailoring and for whom. Given the wide range of possibilities, an important step in the development process is to have focus groups with the goal of hearing from individuals with lived experience about their wants and needs. Through pilot-testing, intervention design and refinement can be an iterative process wherein individuals of the target audience engage with the program, feedback is elicited, and changes are made in response to that feedback. This user-centered approach fits well within the larger preparation phase of a Multiphase Optimization Strategy. Following such development, digital tools should be scientifically tested and optimized, leading to a randomized controlled trial to examine their efficacy in achieving the outcomes of interest (eg, reduction in depression symptoms). This testing phase is necessary to establish a program as evidence-based, which would allow it to stand out in an otherwise large pool of digital applications that are not backed by research.

#### Leveraging New Trial Designs

New trial designs, such as sequential multiple-assignment randomized trials, microrandomized trials, and factorial designs, will be useful in intermediate stages to parse issues such as dosing, sequencing, and personalization. For example, research shows that at least 6 weeks are required for new physical activity habits to form [169]; thus, interventions that are, on average, 8 weeks long lead to more lasting changes than shorter ones [169]. Moreover, it remains unclear whether longer treatments, such as ≥24 weeks, have a greater impact on the general population [170]. It is unknown what duration would be sufficient for various clinical populations to observe changes in both the target behavior and in downstream symptoms and for whom extended support would be necessary. Individual components such as the aforementioned tailoring elements or the inclusion of coaching can also be efficiently tested using these new study designs [170].

#### Understanding the Role of Human Support

Previous work has shown that supervised exercise tends to have a larger impact on anxiety outcomes than unsupervised prescriptions [171]. This parallels guidance from experts in digital mental health that including human support (eg, a lay coach or therapist) alongside internet- or app-based cognitive behavioral and other therapies should enhance retention, engagement, and outcomes. Possible explanations could be greater accountability, the presence of social support, clearer guidelines, opportunities to ask for clarification, in-the-moment personalization, problem-solving, direct affirmation or reinforcement, and a more regular routine. However, guidance does not unilaterally improve outcomes for all digital interventions or all patients [164,172]. There are currently no evidence-based guidelines for implementing human support in digital interventions (ie, when, how often, how much, by whom, and for which users), let alone a nuanced understanding of *how* coaches can effect positive change in adherence or response [173]. Understanding the mechanisms of action would allow developers to maximize automation and most efficiently deploy human support when needed. As human support is the most expensive and scarce resource in digital health solutions, it will be critical to determine how to automate some of these supportive pathways and how to most efficiently identify who needs human support and at what dose.

### Considering Individual Factors

Across intervention types, we must also consider the individual factors that may serve as facilitators of or barriers to engagement and success. This is doubly important for smartphone-based physical activity interventions as there are potential barriers inherent in both smartphone use and physical activity uptake.

#### Barriers to Physical Activity

Research examining barriers to physical activity in those with mental health conditions suggests that individuals with high symptom severity and low self-efficacy may be particularly disinclined to pursue physical activity–based interventions [174,175]. Lack of social support, lack of available time, and fear of injury were also frequently mentioned barriers in a sample of adults with anxiety and depression [174]. In addition, individuals with higher or lower levels of baseline physical activity or fitness may face different barriers and have different needs. A qualitative review suggested that those with lower baseline physical activity wanted an app that had more of a coaching role, whereas those with a higher baseline physical activity preferred an app that helped them intensify or optimize their current physical activity level [126]. In addition, developers should consider the accessibility of exercise suggestions; for example, exercise prescriptions that necessitate equipment, a gym membership, or access to a safe outdoor space may not be generalizable to many otherwise well-suited recipients.

#### Barriers to Digital Mental Health Use

A recent review by Borghouts et al [176] examining barriers to and facilitators of user engagement found that scoring high on neuroticism and agreeableness was associated with greater interest in using smartphone apps to reduce stress, whereas scoring high on extraversion was a predictor of preferring in-person services to web-based options [177]. The severity of baseline symptoms—both psychiatric and comorbid medical concerns—may also play a role in engagement and adherence. Most smartphone-based digital physical activity interventions have been investigated in those with mild to moderate symptoms, which can hamper engagement with apps [178,179]. Some studies suggest that those with mild depression may actually be at an even greater risk of dropout than those with moderate depression [178,180]. Researchers should also be cautious about potential iatrogenic app components. For example, the tracking components inherent in many smartphone-based physical activity interventions, particularly those related to physical health and tracking activity, run the risk of becoming compulsive or rigid. This could pose an issue for individuals with obsessive-compulsive, anxiety, and related disorders that are often characterized by perfectionism or inflexibility. Furthermore, peer support groups within apps, although often helpful, could also lead to negative social comparisons, thus exacerbating depression.

A possible mitigating approach to this would be to introduce smartphone-based physical activity interventions through a stratified care model in which individuals are allocated to different levels of an intervention depending on their clinical needs. In this model, providers could use patient-level data to decide whether an individual would benefit from the smartphone tool as a stand-alone intervention, as a coached version integrated with another level of care (eg, psychotherapy with a clinician), or delivered after progress with another intervention has been made (eg, medication stabilization).

#### Barriers Related to Technology

For many, significant barriers may include technology literacy and access. Multiple studies have identified technology literacy as an obstacle to digital physical activity intervention uptake [126,181] and for digital mental health use [178,180]. This, coupled with the lack of technical support provided by many apps, means that individuals who may be motivated to engage with smartphone-based physical activity interventions are stymied by the inability to navigate the app or seek help when issues arise. This necessitates a user interface and navigability features that can be understood or customized by a range of age groups and technological ability levels, and furthermore, it emphasizes the importance of accessible, embedded technical support tools for those who need them. Coached or guided tools may be helpful in mitigating this literacy issue but still require users to have the basic skills needed to contact their coaches or guides for help. In addition, there is still a subset (15%) of the US population that does not own a smartphone, many of whom represent communities that could benefit the most from flexible, low-cost, and accessible support options [182]. An even greater percentage of Americans lack a stable internet connection; this statistic is highly stratified along racial lines—8 in 10 White adults report having a broadband connection at home, whereas only 71% of Black adults and 65% of Hispanic adults report the same [183]. This suggests a need for digital physical activity interventions that can be accessible from communal settings, such as local community centers or publicly available fitness facilities.

It should be acknowledged that the vast majority of digital mental health interventions are designed—intentionally or not—with primarily White, Western, educated, industrialized, rich, and Democratic populations in mind. Research investigating the efficacy of both in-person and digital physical activity interventions also suffers from similarly nonrepresentative samples, calling into question which validated strategies are universally beneficial. For example, there is compelling research that physical activity interventions (both in person [184,185] and virtual [186]) are effective for health behavior change in Black Americans; however, other studies investigating the perspectives of Black Americans suggest that many in this community face unique social and structural barriers to physical activity that may not be considered by extant programs [187,188]. Thus, engagement in smartphone-based physical activity interventions by marginalized populations might be impacted by both the perception and reality that many of these apps are not designed with their community or culture in mind. Future research should engage with a range of underserved populations in qualitative research to understand the community and cultural values surrounding physical activity and technology use. This information should then be used to collaboratively design new programs or features or culturally tailor existing tools to meet the needs of a broader audience.

All these limitations support the importance of qualitative work as a future direction when building smartphone-based physical activity interventions for mental health. The research synthesized by Carter et al [126], for example, presents valuable insights into individual-level concerns and emerging trends in patient preferences for components and design of apps. In particular, their identification of 2 key mechanisms through which mobile health use facilitates physical activity (strengthening motivation and changes in self-awareness and strategizing) is an important step in boosting engagement and exploring the mechanisms by which these apps function. Engaging qualitatively with a broad spectrum of stakeholders would also be foundational in improving the issue of representation, thereby supporting the goal that smartphone-based physical activity interventions for mental health are acceptable and efficacious for all.

### Practical Considerations

The unfortunate reality of scientifically validated digital mental health products is that the vast majority do not move beyond their success in the laboratory [173], and those that do make it to market face fierce competition, flagging engagement rates, and a lack of financial means to scale the project. Thus, in developing and testing these promising digital mental health–focused physical activity tools, investigators should integrate elements essential for successful dissemination. One proposed pathway for improving the dissemination and ultimate success of digital mental health tools is to connect consumers through employers or public and private insurance companies, who have indicated a growing interest in expanding services to cover digital mental health. To illustrate, Blue Shield of California is now offering the mindfulness meditation app Headspace to subscribers [189]; Cigna offers the mental health app Ginger as part of its service package [190]; and Kaiser Permanente supports the use of Ginger, Calm, and MyStrength [191]. Physical activity technologies that track and manage exercise and step count are even more prevalently covered by insurance. Blue Cross Blue Shield, United, and others have partnered with Fitbit to offer low-cost wearable devices and use of their apps to promote health behavior change. Aetna and Cigna offer similar programs and occasional incentives to people who use health-tracking apps and devices. Taken together, the enthusiasm for apps and devices promoting physical health, as well as the recent foray by insurers into the digital mental health space, suggests that smartphone-based physical activity interventions for mental health may be prime for scalable coverage. This also means that academics developing such tools should be mindful when designing research studies to collect outcome data relevant to insurers and other payers, such as outcomes related to health care costs (reduction in insurance claims and physician or therapist visits), disability-adjusted life years (reduction in overall illness burden), adoption and engagement rates, and user data such as acceptability and fidelity (whether people use the tools as intended). In addition, investigators and designers should carefully consider the costs inherent to their interventions, such as relying on “off-the-shelf” versus research-grade devices and other platforms, the extent to which an intervention relies on human support to be administered, and the broader infrastructure required for implementation and sustainment, all of which will alter accessibility and scale. By designing research studies on smartphone-based physical activity interventions with true scalability in mind, researchers will be better poised to expand their intervention beyond academia and better achieve the goal of connecting evidence-based interventions with those who need them.

### Conclusions

Physical activity has well-known and broad mental health benefits. However, a minority of at-risk individuals or those with mental disorders meet even the minimum exercise recommendations. Smartphones may bridge this gap given their pervasiveness in daily life, capacity to help concurrently manage multiple dimensions of personal health, and ability to engage key mechanisms of behavior change. Although early data for smartphone-based physical activity interventions reducing psychological symptoms are encouraging, overall, surprisingly little work has been done in this area. Therefore, there is untapped potential for developing and disseminating accessible, beneficial tools that can have a great public health impact.

## Abbreviations

CBT: cognitive behavioral therapy
HIIT: high-intensity interval training
